# Poor adult nutrition impairs learning and memory in a parasitoid wasp

**DOI:** 10.1038/s41598-021-95664-6

**Published:** 2021-08-10

**Authors:** Hossein Kishani Farahani, Yasaman Moghadassi, Jean-Sebastien Pierre, Stéphane Kraus, Mathieu Lihoreau

**Affiliations:** 1 Equipe Recherches Agronomiques, Agronutrition Co, Carbonne, France; 2 Department of Plant Protection, Faculty of Agriculture and Natural Resources, University of Tehran, Karajs, Iran; 3 Rennes 1, UMR-CNRS 6553 EcoBio, University of, Avenue du Général Leclerc, Campus de Beaulieu, 35042 Rennes Cedex, France; 4 Research Center On Animal Cognition (CRCA), Center for Integrative Biology (CBI), CNRS, UMR 5169 CNRS, University of Toulouse III, Toulouse, France

**Keywords:** Cognitive neuroscience, Feeding behaviour, Learning and memory, Neuronal physiology

## Abstract

Animals have evolved cognitive abilities whose impairment can incur dramatic fitness costs. While malnutrition is known to impact brain development and cognitive functions in vertebrates, little is known in insects whose small brain appears particularly vulnerable to environmental stressors. Here, we investigated the influence of diet quality on learning and memory in the parasitoid wasp *Venturia canescens*. Newly emerged adults were exposed for 24 h to either honey, 20% sucrose solution, 10% sucrose solution, or water, before being conditioned in an olfactory associative learning task in which an odor was associated to a host larvae (reward). Honey fed wasps showed 3.5 times higher learning performances and 1.5 times longer memory retention than wasps fed sucrose solutions or water. Poor diets also reduced longevity and fecundity. Our results demonstrate the importance of early adult nutrition for optimal cognitive function in these parasitoid wasps that must quickly develop long-term olfactory memories for searching suitable hosts for their progeny.

## Introduction

Animals rely on various forms of learning and memories to exploit resources in their environment and adapt to changing conditions^1–3^. These cognitive abilities are sustained by brains that require large amounts of proteins to grow^4^, but also lipids and carbohydrates for maintenance^5–7^. The process of learning, itself, imposes important energetic costs^8^, and the formation of persistent (long-term) memories involves protein synthesis^9,10^. Therefore, the ability of animals to acquire key nutrients in food is expected to directly impact their cognitive performances^11^.

Malnutrition is known to affect cognitive functions in vertebrates (e.g. pigeons:^12^, mice:^13^, cats and dogs:^14^). In humans, for instance, high fat and caloric diets have been associated with hippocampal-dependent memory loss^15,16^. By contrast, little is known about the cognitive effects of malnutrition in invertebrates. Insects, in particular, rely on an impressive range of learning and memory forms to interact socially and forage, and these cognitive abilities are implemented by only few neurons^17,18^. The miniature brain of insects is thus particularly vulnerable to a range of environmental stressors, including poor nutrition^19^. Recent studies showed negative impacts of poor diets. For instance, in the fruit fly *Drosophila melanogaster*, larvae fed diets with unbalanced protein to carbohydrate ratios showed reduced learning performances in an aversive olfactory differential learning task^20^. In the Western honey bee *Apis mellifera*, adults fed pollen with a deficit in specific fatty acids (i.e. Omega-3, Omega-6) had impaired learning and memory performances in an appetitive olfactory differential learning task^21,22^. These cognitive effects of malnutrition may incur particularly strong fitness costs in many solitary species where adults rely on learning and memory to find food and nourish their progeny by themselves.

Solitary parasitoid wasps, such as *Venturia canescens* (Hymenoptera: Ichneumonidae), learn to associate an odor with a high-quality host to select nutritionally rich environments for the development of their offspring^23–27^. In this synovigenic species, females continue to produce and mature eggs throughout adult life. Females thus need to regularly acquire nutrients for egg production. Before engaging in oviposition, these wasps encompass a critical period soon after emergence, where they need to find food^28–31^. Males and females typically acquire carbohydrates from nectar and honeydew^32–35^. Other key nutrients such as proteins, minerals and fat, are occasionally obtained from pollen^36^. Since olfactory memory formation is nutritionally demanding, we hypothesized that wasps fed highest quality diets would show the best cognitive performances.

Here, we tested whether diet quality during early adulthood affect the cognitive performances of *V. canescens* wasps, a thelytokous (i.e. a type of parthenogenesis in which unfertilized eggs produce females), koinobiont (i.e. the host continues to feed and grow after parasitization) and solitary endoparasitoid of lepidopterous larvae^28,37,38^. We experimentally exposed emerging females to one of four nutritional conditions of decreasing quality in terms of nutrient concentration, nutrient diversity, and energy content: honey (see composition in Table 1), sucrose 20%, sucrose 10%, and water. We then tested the impact of diet on cognition in a conditioning assay in which wasps had to associate an odor to a host reward (larvae of *Ephestia kuehniella*) in a flight tunnel. We further tested the influence of diet on fitness by monitoring the longevity and the reproductive success of these wasps. Details about sample sizes used in each experiment are available in Table 1.

**Table 1:** The numbers of wasps used and actually tested for each experiments.

Experiment	Treatment	Wasp status	Initial No. of wasps	No. tested females
Learning	Honey	Conditioned	60	50
	Sucrose 20%	Conditioned	60	50
	Sucrose 10%	Conditioned	60	50
	water	Conditioned	60	50
Memory retention	Honey	Conditioned	720	720
	Sucrose 20%	Conditioned	720	300
	Sucrose 10%	Conditioned	720	450
	water	Conditioned	720	250
Longevity	Honey	Conditioned	30	30
		Unconditioned	30	30
	Sucrose 20%	Conditioned	30	30
		Unconditioned	30	30
	Sucrose 10%	Conditioned	30	30
		Unconditioned	30	30
	Water	Conditioned	30	30
		Unconditioned	30	30
Fecundity	Honey	Conditioned	30	30
		Unconditioned	30	30
	Sucrose 20%	Conditioned	30	30
		Unconditioned	30	30
	Sucrose 10%	Conditioned	30	30
		Unconditioned	30	30
	water	Conditioned	30	30
		Unconditioned	30	30

## Results

### Wasps did not show innate odor preference

**Figure 1:**
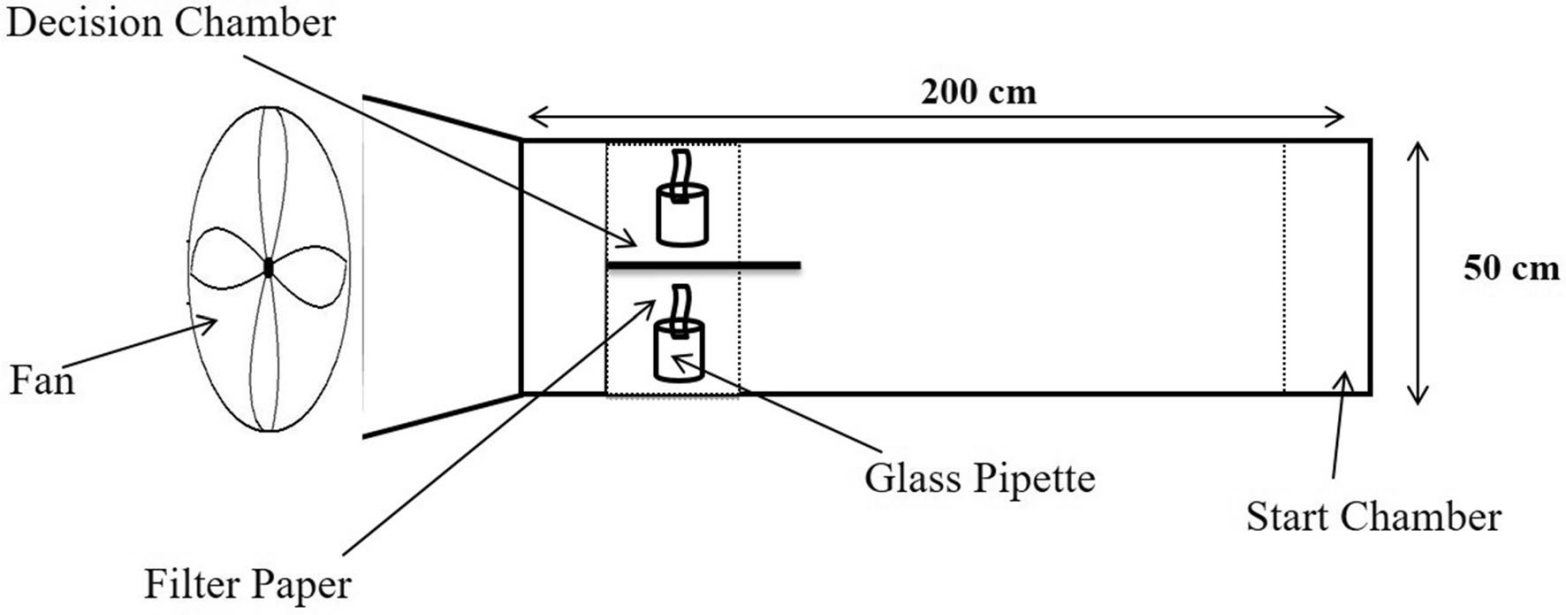
Schematic view of the flight tunnel (top view). Individual wasps were introduced in the start chamber and observed choosing between the two odors displayed on filter papers in the decision chambers for 15 min.

We first tested the influence of the nutritional condition on innate attraction to odors, by giving individual wasps a simultaneous choice between two odor sources (orange and vanilla) in a flight tunnel with two decisions chambers for 15 min (see details in Fig. 1). The wasps did not display any preference for either odor, irrespective of their nutritional condition (*χ*_*2*_^2^ = 0.13, *P* = 0.93, N = 50). The proportion of wasps that made no choice (i.e. when the wasps did not fly after 5 min in the tunnel) remained stable (30 ± 2%) and was similar across nutritional conditions (*χ*_*2*_^2^ = 0.9, *P* = 0.69, N = 50). This indicates that, at our experimental scale, the nutritional condition did not affect the motivation nor the motor activity of wasps in response to odorants. We therefore arbitrarily chose the orange odor as the conditioned odor (CS +) and the vanilla odor as the new odor (NOd) in all the subsequent behavioral tests.

### Wasps fed honey showed highest learning performance

**Figure 2:**
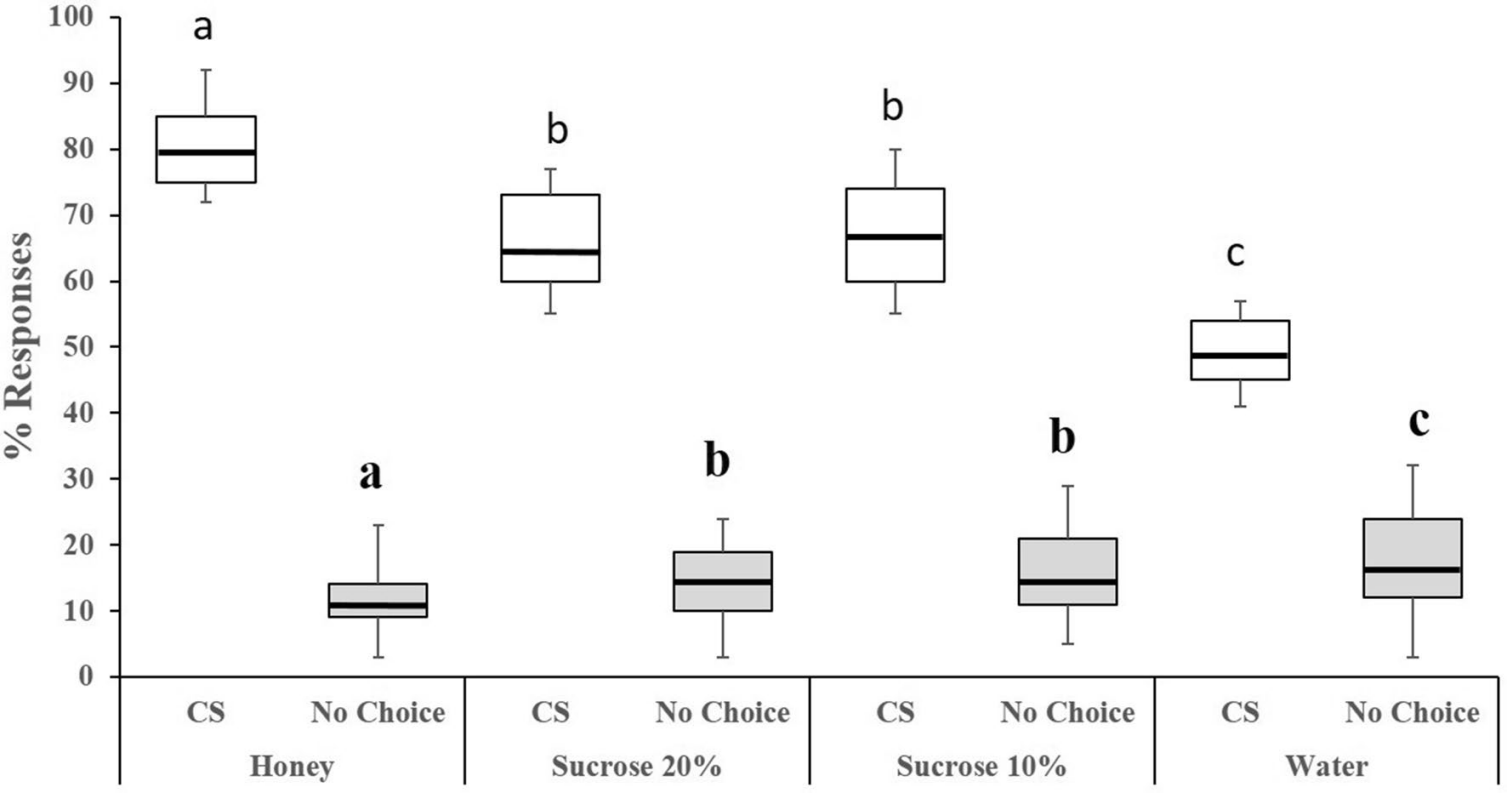
Learning**.** Percentages of correct choices for the conditioned stimulus (CS, orange odor) and no choices (% not responding individuals) for each nutritional condition. In the boxplot, the central line is the median, the edges of the box are the 25th and 75th percentiles, the whiskers extend to the most extreme data points not considered outliers. Outliers are represented by points. Median responses are indicated for each group in the learning performance graph. Generalized Linear Models (GLM) were implemented with the binomial family error and logit link. Different letters above bar plots indicate significant differences between the treatments after Bonferroni correction (*P* = 0.0125). N = 50 wasps per nutritional condition (200 wasps in total).

We then tested the effect of the nutritional condition on olfactory learning. To do so, we first conditioned each wasp in the presence of 30 host larvae and the orange odor (CS +) for 2 h. We then tested the conditioned wasps for odor preference by giving them a choice between the CS (orange) and the NOd (vanilla) in the flight tunnel for 15 min. We ran behavioural assays on groups of 10 wasps for each diet every day. Learning was observed in wasps exposed to each of the four nutritional conditions but at different magnitudes (Fig. 2; Binomial GLM Feeding; *χ*_*1*_^2^ = 87.33, P < 0.0001; Conditioning: *χ*_*1*_^2^ = 14.48, *P* = 0.0001; Feeding × conditioning: *χ*_*1*_^2^ = 7.7, *P* = 0.005). The highest proportion of correct choices for the CS (mean proportion of wasps choosing the CS per day: 85 ± 3%, N = 50) was observed in wasps fed honey. This proportion decreased with diet quality, reaching intermediate levels in wasps fed sucrose diets (72 ± 2%, N = 200), and a minimum level in starved wasps (62 ± 2%, N = 200). The proportion of wasps that did not make a choice remained low across nutritional conditions (12 ± 2%, N = 200; Fig. 2), but increased with decreasing diet quality, reaching a maximum in starved wasps (29 ± 3%, N = 200; Binomial GLM Feeding; *χ*_*1*_^2^ = 12.3, *P* = 0.006; Conditioning: *χ*_*1*_^2^ = 8.93, *P* = 0.002; Feeding × conditioning: *χ*_*1*_^2^ = 7.61, *P* = 0.005; Fig. 2). Therefore, wasps fed highest diet quality showed highest learning performances. To control for any motivational effect of the nutritional condition on learning, we compared the latency before wasps made a choice. We found a significant effect of conditioning (F = 19.12, P < 0.0001), but no effect of the nutritional condition (F = 3.46, *P* = 0.064), or the interaction between the two (F = 0.16, *P* = 0.069). Thus, differences in learning performances were not due to differences in locomotion or motivation.

### Wasps fed honey showed longest memory retention

**Figure 3:**
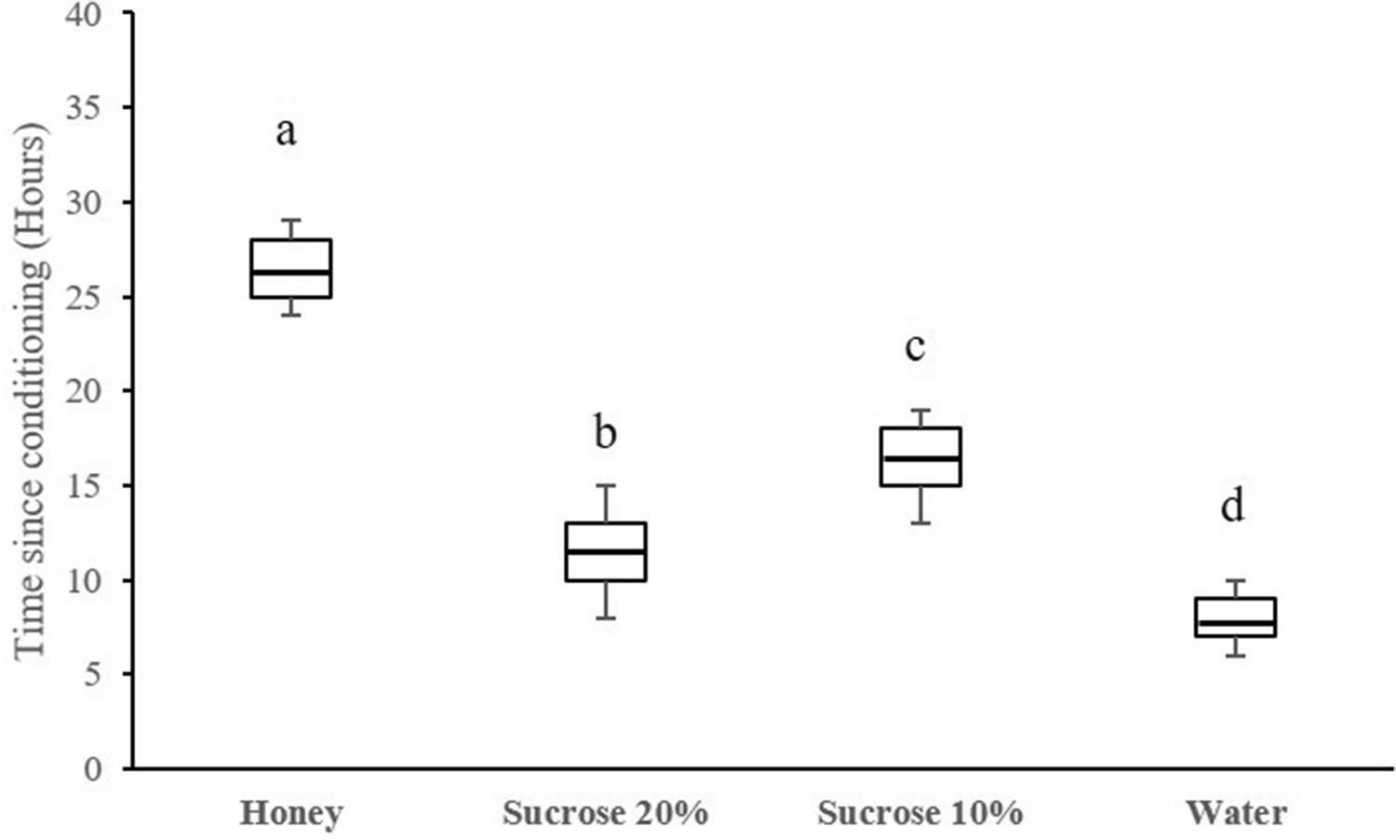
Memory retention times. In the boxplot, the central line is the median, the edges of the box are the 25th and 75th percentiles, the whiskers extend to the most extreme data points not considered outliers. Outliers are represented by points. Median memory retention time is indicated for each group in the memory graph. Memory retentions were compared using one way analysis of variance (ANOVA). Different letters indicate significant differences between the treatments after Bonferroni correction (*P* = 0.0125). N = 50 wasps per nutritional condition and time interval (2400 wasps in total).

We further tested the effect of the nutritional condition on olfactory memory retention by testing wasps in the flight tunnel at different time periods, between 2 and 30 h post conditioning (Fig. 3). Memory retention was significantly longer in wasps fed honey (27 ± 3 h, N = 600) than in wasps fed 20% sucrose solution (12 ± 3 h, N = 600), or 10% sucrose solution (17 ± 2 h, N = 600), or in wasps fed water (8 ± 2 h, N = 600; ANOVA: F = 302.2, P < 0.0001). Therefore, wasps fed highest diet quality showed longest memory retention. Note however that wasps fed 10% sucrose solution had a significantly longer memory retention than wasps fed 20% sucrose solution (Fig. 3), suggesting high variability in the behavioural responses.

### Wasps fed honey had highest longevity and fecundity

**Figure 4:**
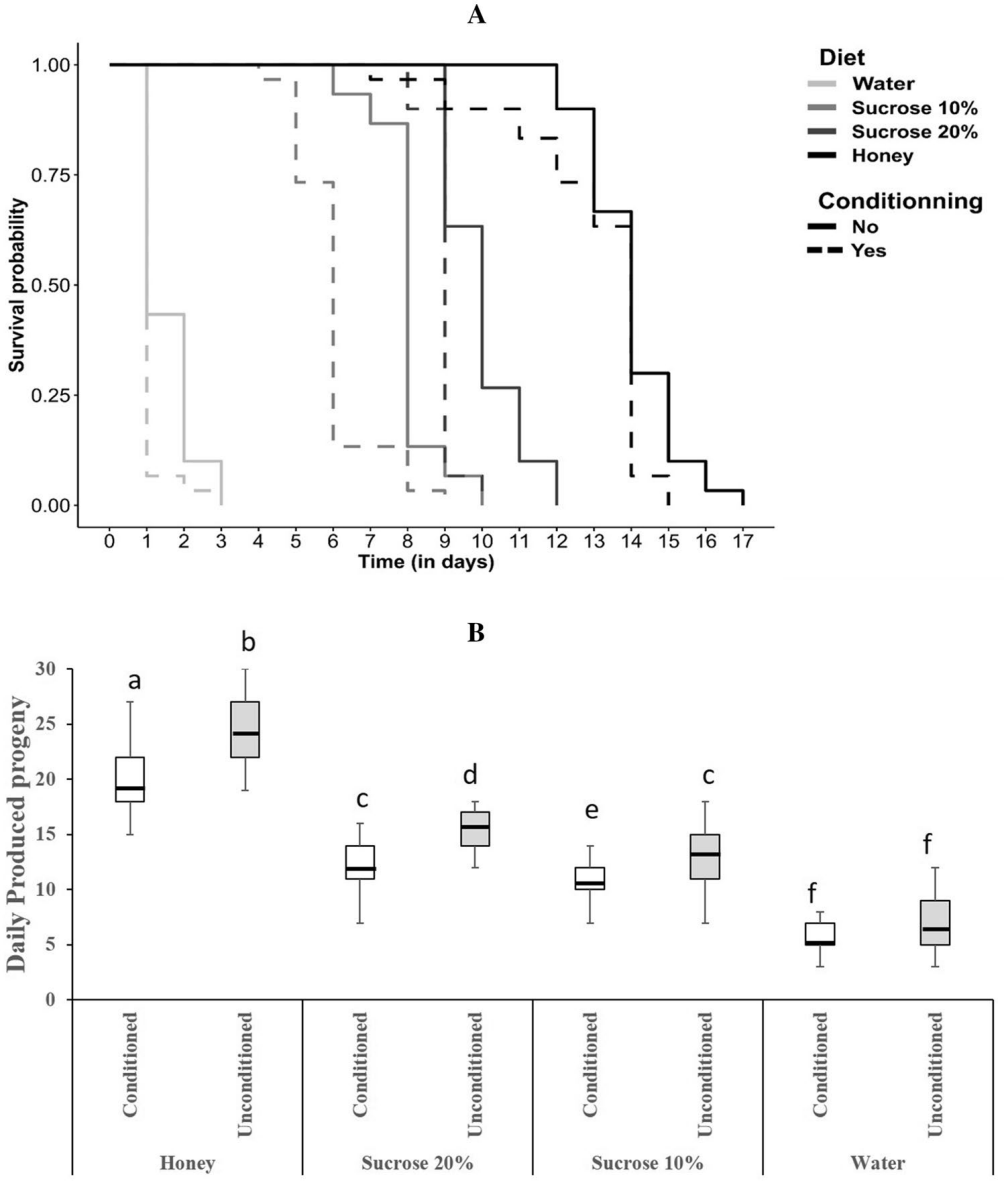
Longevity and fecundity. Effect of the nutritional condition (colors) and conditioning (solid or dashed lines) on survival probability (**a**) and fecundity (**b**) of female wasps. Survival curves were obtained from Kaplan Meier model (function survfit in R package “survival” (Therneau 2015)). In the boxplot, the central line is the median, the edges of the box are the 25th and 75th percentiles, the whiskers extend to the most extreme data points not considered outliers, outliers are represented by points. Median lifespan is indicated for each group in the fecundity graph. Different letters indicate significant differences between the treatments after Tukey post-hoc.

We finally tested the effects of the nutritional condition (i.e. diets) and conditioning (i.e. conditioned vs. unconditioned wasps) on fitness, by measuring the longevity and the fecundity of wasps (N = 30 per group). Both the nutritional condition and conditioning had significant effects on longevity (Fig. 4 A; Cox model; nutritional condition: p < 0.001; conditioning: p < 0.001; nutritional condition x conditioning: *P* = 0.504). Honey diet reduced the risk of death by 16 compared to water (Hazard Ratio = 0.06), while conditioning increased this risk by 3 (Hazard Ratio = 2.97) indicating a cost of learning and memory formation. The nutritional condition also influenced the fecundity of wasps, so that honey diet increased by 2.77 the number of offspring per female in comparison to water, as well the conditioning experience reducing the production of progeny by 0.9 (Fig. 4 B; Poisson GLM; diet: χ2 = 426.85, p < 0.001; χ2 = 11.70, conditioning: p < 0.001; diet x conditioning: χ2 = 1.86, *P* = 0.6). Thus overall, early nutritional experience had long lasting effects on adult fitness.

## Discussion

Recent studies showed that a lack of specific nutrients^21,22^ or an unbalanced ratio of these nutrients^20^ in food can result in impaired cognitive abilities in model insect species, such as honey bees and fruit flies. Here, we found that olfactory learning performances and memory retention times of *V. canescens* wasps were considerably affected by a poor diet soon after adult emergence. High quality nutrition might be critically important for parasitoid females in general, as they that must learn to locate and recognize hosts in order to lay their eggs in favorable environments. Any impairment of such abilities can incur important fitness costs.

*V. canescens* can learn a variety of olfactory and visual stimuli associated to their hosts^25,39^. Here we found that a poor diet (sucrose solutions, or water) at early stages of adulthood significantly reduced olfactory learning and memory retention times. These effects are not developmental since wasps were exposed to nutritional treatments during 24 h as adults only. Impaired cognition therefore likely reflects physiological needs for cognitive function in fully developed brains. The fact that conditioning affected longevity, irrespective of the diet, reveals physiological costs associated to learning and memory formation, as previously reported in fruit flies^9^.

Unsurprisingly, wasps exposed to food (either honey or sucrose solution) always performed better than wasps provided only water, that presumably lacked energy for basal brain functions. But how can we explain differences in wasps fed honey or sucrose solutions? The fact that wasps exposed to different diets did not differ in their proportions of no choices, indicates that food composition did not affect the locomotion or the motivation of the wasps. This was confirmed with an absence of difference in the latency to make a choice in the leaning experiments. Therefore, the observed effects are specific to learning and memory. We discuss two, non-mutually exclusive, possible mechanisms.

Firstly, honey could yield higher cognitive performance because it contains more total nutrients and/or energy. Overall, honey contains much more total energy (284 cal/mg) than 20% sucrose solution (80 cal/mg) and 10% sucrose solution (40 cal/mg). It contains monosaccharaides, like glucose and fructose, that can directly or indirectly (when in combined forms such as glycogen) be absorbed^40,41^. These monosaccharaides constitute the carbohydrate-based energy source for the insects. By contrast, sucrose is a disaccharide consisting of one glucose and one fructose molecule that must be broken down by enzymatic reactions before being used as energy source, thus constituting an additional physiological cost^41^. This difference in the total amount of carbohydrates in diets may also explain the important difference in survival by wasps fed honey and wasps fed sucrose solution, as carbohydrates have a well-known positive impact on longevity in insects^32,33,42^.

Alternately, the effects of early adult nutrition on cognition may be due to the lack of specific nutrients in food. Honey contains a rich diversity of nutrients including amino acids and minerals that were not present in sucrose solutions (e.g. Table 2). The most abundant amino acid in honey is proline^43–45^. The endogenous neutral amino acid L-proline exhibits a variety of physiological and behavioral actions in the nervous system and in increasing or improving memory retention in vertebrates^46,47^. In honey bees, a decrease of proline in body led to lower learning ability and memory retention^46^. In our experiments, the lack of proteins in sucrose solution likely explains the reduced reproductive success of wasps fed sucrose compared to wasps fed honey, as these are required nutrients for egg production. Honey also includes several macro and micro-elements minerals such as potassium, magnesium, calcium, iron, phosphorus, sodium^48^. Potassium is the most abundant mineral^49^. In human, potassium uptake increases learning and memory^50^. Honey also contains potassium and sodium. The Na + /K + -pump on postsynaptic receptors plays a critical role in synaptic transmission in the brain and a lack of these elements may induced impaired cognitive functions^51–53^.

**Table 2:** Honey composition. Honey analysis was made by ASA Laboratory (Tehran, Iran) based on ISIRI-7610, ISIRI-92 and European Honey Directive and the Codex Alimentarius Standard for Honey standards.

Ingredients	Per 100 g
Protein	1.5 g
Carbohydrates	70 g
**Sugars**	
Glucose	30 g
Fructose	38 g
Fat	0
Fiber	1 g
**Vitamins**	
B6	4%
C	3%
Riboflavin	8%
Folate	3%
Potassium	50 mg
Sodium	10 mg
Total Energy	284 cal/mg

Our experimental design does not allow to disentangle these mechanisms. Nonetheless, our results demonstrate the crucial importance of adult feeding on their cognitive abilities, longevity and reproduction. These observations in the lab suggest poor adult nutrition can have dramatic consequences for wasps in natural conditions. In fact, the importance of nutrition may be greatly magnified in the wild, where wasps must develop costly learning and memory to identify suitable hosts for oviposition, but also need to locate these hosts using olfactory cues associated to the presence of larvae^54^. Our population of wasps originates from outdoor pomegranate orchards in dried areas in which we believe insects suffer from food and host limitation. It is therefore very likely that wasps must fly long distances in order to parasitize hosts, thereby incurring additional energetic costs of movements^55–57^. The importance of learning is reflected by the fact that wasps are capable of developing long-term memories, lasting up to 30 h, after a single exposure to the odour and the host. This confirms a previous study in *V. canescens*^58^ and agrees with a recent report in honey bees^59^ where appetitive olfactory learning is also critical for efficient foraging.

Nectar is an important source of food for parasitoids. It is composed of simple sugars in solution whose content can vary from 15 to 75% by weight^60^. The three common sugars are glucose, fructose, and sucrose, but traces of various oligosaccharides (e.g., raffinose) are sometimes present^61^. The sugar content of nectar is relatively similar to that of honey we used in our experiments (see Table 2). It is thus very likely that nectar also positively influences olfactory cognition in female wasps. In these conditions, feeding of high quality foods, such as nectar, may provide considerable fitness advantages to wasps. Future experiments could further explore this critical interaction between diet and cognition, using experimental designs of nutritional ecology based on artificial diets controlling for the amount and concentration of nutrients (e.g.^11^). In recent years these approaches have been very successful to identify the effects of specific nutrients and energy contents on fitness traits in many organisms (e.g. flies:^32,33^; crickets:^42^; mice:^57^), including hymenoptera (e.g. honey bees:^62^; bumblebees:^63^), and yield considerable promises for investigations in cognition research.

## Material and methods

### Insect culture

Wasps (*V. canescens*) and their hosts (flour moth *Ephestia kuehniella* (Lepidoptera: Pyralidae)) were cultured and tested in incubators at 25˚C with a 16:8 Light: Dark photoperiod and 50 ± 5% relative humidity. The *V. canescens* culture originated from 70 wild caught individuals sampled in 2017 (Saveh, Markazi province, Iran) and maintained at the University of Tehran. Natural populations of *V. canescens* contain both thelytokous (asexual) and arrhenotokous (sexual) individuals^64^. We only used thelytokous wasps as they are dependent on nutritional resources acquired as adults (income resources) for reproduction and survival^29^. *E. kuehniella* eggs were obtained from a laboratory culture at the Insectary and Quarantine Facility of the University of Tehran. *E. kuehniella* larvae were reared on a standard diet made of 48.5 g of wheat flour and 3 g of brewer yeast^25^.

To obtain experimental individuals, groups of 30 one-day old female wasps were presented ca. 200 5th instar host larvae in a large plastic box (30 × 20 × 20 cm) and allowed to lay eggs for 24 h^25^. Groups of twenty parasitized host larvae were kept in smaller boxes (5 × 5 × 3 cm) until the emergence of adult wasps. The parasitized hosts were maintained under controlled condition, 25˚C with a 16:8 Light: Dark photoperiod and 50 ± 5% relative humidity.

### Nutritional conditions

One day old female wasps were isolated in glass boxes (10 × 5 × 3 cm) and given ad libitum access to either: (1) honey (70% carbohydrates: fructose (38% w/v), glucose (30% w/v), 1% fibers, 1.5% protein, total energy (284 cal/mg)) (see details in Table 2), (2) 20% sucrose solution (w/v, total energy(80 cal/mg)), (3) 10% sucrose solution (w/v, total energy (40 cal/mg)), (4) or water (to avoid dehydration). Wasps were provided honey as droplets on wax-coated strips of paper. Sucrose solutions were provided in gravity feeders (i.e. 4 cm^3^ plastic capsule with a capillary tube inserted at the bottom). During the experiments, the wasps were regularly monitored to make sure that they can access food easily. Wasps were kept in these boxes for 24 h before the behavioral assays.

### Behavioral assays

We performed the cognitive tests in a flight tunnel (200 × 50 × 50 cm) made of transparent Plexiglas (Fig. 1; for more details see^65^). The experimental room was illuminated with 2000 lx lights provided by LED lights (Pars Shahab Lamp Co., Iran)^25^. Air was driven through the flight tunnel by a fan located at the upwind end, and extracted outside by a fume hood at the downwind end (wind speed of 70 cm/s). The end opposite to the start zone of the tunnel was divided by a glass separator wall in two decision chambers. Each decision chamber contained an odorant stimulus presented on a filter paper attached to a glass pipette placed vertically on a stand. The behavioral data were recorded though visual observation by an experimenter blind regarding to the nutritional conditions of the wasps.

### Innate odor preference

To control for any effect of the nutritional condition on odor preference, we assessed the innate odor preference of the wasps. Fifty one day old wasps from each nutritional condition were given a simultaneous choice between two synthetic odors in the flight tunnel: orange and vanilla (97% pure odors: Adonis Gol Darou Group, Iran)^66^. We assumed that our wasp population has never been exposed to these odors prior to the tests, neither in the field nor in the lab. Each odor was presented on a filter paper scented with 1 μl of the solution in one of the decision chambers of the tunnel. The wasp was placed at the start zone of the tunnel and allowed to make a choice between the two decision chambers for 15 min. Any wasp that spent more than three consecutive minutes within 3 cm around the scented filter paper (landed, walking or hovering around) was considered as “making a choice”. Previous studies show that a wasp landing on an odor site for more than three minutes remains longer than 15 min on that site^25^. Any wasp that did not fly in the tunnel within 5 min after the beginning of the test was considered as “making no choice”^25^. After each assay, the flight tunnel was cleaned with ethanol (70% V/V). Odors were swapped every five assays. Fifty wasps were tested for each nutritional condition (N = 200 wasps in total). Because we found no innate attraction for either odors, we arbitrarily selected the orange odor as the conditioned stimulus (CS +) and the vanilla odor as the new odor (NOd) in all subsequent experiments.

### Learning

We assessed the effect of diet on learning performances using olfactory conditioning. To make sure the wasps had some oviposition experience, and thus avoid the inter-individual variability in the sequence and duration of behavioral events associated with learning from the first host encountered^67^, female wasps were individually exposed to 15 host larvae (5th instar) for 15 min in a vial (2 cm × 10 cm) before conditioning. Sixty of these wasps were then transferred into conditioning tanks (25 cm × 25 cm × 25 cm) with another 30 host larvae (5th instar). The orange odor (CS +) was pumped into the tanks at an air speed of 1 m/s. The wasps were maintained in these conditions for 2 h during which they could associate the orange odor to the presence of host larvae. As some females occasionally died, were lost, or failed to oviposit during the procedure, 50 out of the 60 wasps conditioned were selected to be monitored in the flight tunnel (see details in Table 2).

Learning performance was assessed 15 min after conditioning by presenting the odors of orange (CS) and vanilla (NOd) in each decision chamber of the flight tunnel. Every wasp that spent more than three consecutive minutes within 3 cm of the CS was considered as making a “correct choice”. Wasps that spent more than three minutes within 3 cm of the NOd made an “incorrect choice”. Wasps that did not fly within 5 min after the beginning of the test made “no choice”. Fifty wasps were tested for each nutritional condition (N = 200 wasps in total). Latency time, the time that wasps need to make a choice in flight chamber, was recorded, this recording was started when wasps passed the start chamber and ended when they made a choice as described.

### Memory retention

We tested the effect of nutritional condition on memory retention time by observing the responses of the conditioned wasps either 2 h, 4 h, 6 h, 8 h, 10 h, 12 h, 14 h, 16 h, 18 h, 20 h, 24 h or 30 h after conditioning^58^. The responses of the wasps to the CS and the NOd were recorded in the flight tunnel as previously described (see section learning). Fifty wasps were observed in each of the four nutritional conditions and twelve time intervals (N = 2400 wasps in total). These new wasps were conditioned as described before. All memory tests were carried out during the day.

### Longevity and fecundity

We tested the effect of the nutritional condition and conditioning on fitness by measuring the longevity and fecundity of conditioned and unconditioned wasps in the four nutritional conditions. New wasps were conditioned for these experiments. To study longevity, we maintained the wasps individually on one of the four nutritional conditions in a plastic box (30 × 20 × 20 cm). We recorded the number of dead wasps every day until all wasps died (18 days). To study fecundity, we placed each wasp in an oviposition cage with 30 host larvae (5th instar). Host larvae were removed daily and kept in small boxes (5 × 5 × 3 cm) until the emergence of adult wasps under controlled condition, 25˚C with a 16:8 Light: Dark photoperiod and 50 ± 5% relative humidity. Every day, we monitored the number of wasps emerging from the parasitized hosts. Thirty females were used for each combination of conditioning and nutritional condition for longevity and for fecundity (N = 480 wasps in total).

### Statistical analysis

We analyzed the innate odor responses and learning data using SAS (SAS Institute Inc. 2003). Information about the number of wasps used and tested in each experiments are available in Table 1. We compared the innate odor response of wasps exposed to different nutritional conditions using Chi-square tests. We tested the effect of the nutritional conditions (diets) and conditioning on learning of conditioned odors using a Generalized Linear Model (GLM) implemented in the procedure GENMOD (binomial family error, logit link function). Also latency time was analyzed using Cox proportional hazard models. We compared the least square estimates of the proportions in each level using the Chi-square approximation. When we found a significant effect of the treatment, we applied a Bonferroni’s post hoc multiple comparison tests, and evaluated the two-by-two comparisons at the Bonferroni-corrected significance level of *P* = 0.05/k, where k is the number of comparisons.

We estimated the effect of the nutritional condition on memory retention by developing a dynamic and statistical model following Kishani Farahani et al.^68^. Briefly, the estimation of forgetting relies on a series of observations recorded at different times t_1_; t_2_; …t_n_ after conditioning. At each time, a set of *n*_*t*_ subjects was subjected to a choice test with three possible responses: *a*; *b*; and *c*, which correspond respectively to a preference for the orange side, a preference for vanilla side, and to a no choice. The forgetting of conditioning results in a switch from a high level to a lower level of correct responses, a simultaneous switch from a low level to a high level of no choices, and a switch from a very low to a moderate level of incorrect choices. A constraint links the three responses as *n*_*a*_ + *n*_*b*_ + *n*_*c*_ = *n*_*t*_ or *n*_*c*_ = *n*_*t*_*—n*_*a*_*—n*_*b*_. The course of these three responses over time can be described by two logistic functions written here as probabilities, *p*_*a*_*, p*_*b*_*, p*_*c*_, constrained by *p*_*a*_ + *p*_*b*_ + *p*_*c*_ = 1:

1 $$p_{a} = k_{a} - \frac{{k_{a} - a_{a} }}{{1 + e^{{\left( { - b_{a} \left( {t - t0} \right)} \right)}} }} + a_{a}$$

2 $$p_{c} = \frac{{k_{c} - a_{c} }}{{1 + e^{{\left( { - b_{c} \left( {t - t0} \right)} \right)}} }} + a_{c}$$

3 $$p_{b} = 1 - p_{a} - p_{c}$$

where *k*_*a*_, respectively *k*_*c*_*,* and *a*_*a*_, respectively *a*_*c,*_ define the asymptotic level and baselines of the logistic models (1) and (2): the baselines are *a*_*a*_ and *a*_*c*_, and the asymptotic level are *k*_*a*_ + *a*_*a*_in model (1), *k*_*c*_ + *a*_*c*_ in model (2). *k*_*a*_ + *a*_*a*_ estimates the initial state in model (1), and *a*_*c*_ the final state. It is the inverse in model (2), where a_c_ is the initial state and *k*_*c*_ + *a*_*c*_ the final state. A supplementary restriction lies in the fact that, as t0 represents the mean time to oblivion, i.e. the inflection time point of the logistics functions; it has to be the same in all three equations. The data consist of a vector of three counts: $V_{t} = \, \left( {n_{at} , \, n_{bt} , \, n_{ct} } \right)$ the respective number of subjects responding a; b or c at time t. An R script was written to do this (see Supplementary text S1). The model defined by Eqs. 1–3 was fitted individually on each set of ten data. The maximization of the likelihood cannot be fully automatic and requires an initial guess of the seven parameters *k*_*a*_*; a*_*a*_*; b*a*; k*c*; a*_c_*; b*_*c*_*; t0*. This was done by a visual evaluation of each graphic representation of the crossed levels. We compared memory retention times across nutritional conditions using an Analysis of Variance (ANOVA, using SAS).

We analyzed longevity and fecundity data in R 4.0.3 (R Core Team 2020). We tested the effect of the nutritional conditions, conditioning and their interactions on longevity using a Cox proportional hazards regression model (function coxph in package “survival”^69^). We tested the effect of the nutritional conditions, conditioning and their interactions on fecundity using generalized linear mixed-effects model (GLMM) with Poisson family (function glmer in package “lme4” ^70^). We added wasp identity and day of experiment as random factors in all models.

### Animal welfare ethics

All methods were carried out in accordance with Iranian and European regulations. Experimental protocols were approved by the University of Tehran. The study was carried out in compliance with the ARRIVE guidelines. The current study was not included any potentially harmful manipulations and invasive samples**.** During the experiments all wasps were reared on flour moth larvae in the laboratory conditions at 25 ˚C with a 16:8 L: D photoperiod and 50 ± 5% R.H. Adult parasitoids were fed on undiluted honey. All wasps were maintained and tested under the same conditions, and throughout all experiments, one day old wasps were fed on a 10% honey solution. All animals were obtained from a culture maintained at the Insectary and Quarantine Facility, University of Tehran. After finishing the experiments all wasps were kept at the same condition and were fed with undiluted honey, and during this period they were exposed to hosts to have routine life stages including feeding and oviposition.

## Supplementary Information

Supplementary Information 1.

Supplementary Information 2.
