# Supplementary material for: Poor adult nutrition impairs learning and memory in a parasitoid wasp
Source: Sci Rep. 2021 Aug 10;11:16220. doi: 10.1038/s41598-021-95664-6 (PMC8355316; doi:10.1038/s41598-021-95664-6)
Supplement: Supplementary file 1 — Supplementary Information 1. [file 41598_2021_95664_MOESM1_ESM.docx]

learn2=read.table("i:/articles en cours/hossein/learning.txt",header=T)

Z=data.frame(matrix(0,10,10));Z

names(Z)=c("ka","aa","ba","kc","ac","bc","t0","se","lower","upper")

rownames(Z)=c("asex1","sex1","asex2","sex2","asex3","sex3","asex4","sex4","asex5","sex5")

#===============================================================================

# llv : the log-likelihood function. beta = vector of seven parameters

#===============================================================================

llv=function(beta,time,x,m)

{

ka=beta[1];aa=beta[2];ba=beta[3]

kb=beta[4];ab=beta[5];bb=beta[6]

t0=beta[7]

n=length(time)

u=ka-(ka-aa)/(1+exp(-ba*(time-t0)))+aa

#print(u)

v=ab+(kb-ab)/(1+exp(-bb*(time-t0)))

#print(v)

w=1-u-v

#print(w)

S=x[,1]*log(u)+x[,2]*log(v)+(m-x[,1]-x[,2])*log(w)

lv=sum(S)

lv

}

#===============================================================================

# End of function llv

#===============================================================================

#===============================================================================

# FUNCTION forgiveFit

# Acts on the vector V of observations

# Calculates the parameters of a forgiveness model

#================================================================================

forgiveFit=function (V=testcase,par=c(ka,aa,ba,kb,ab,bb,t0))

{

par(mfrow=c(1,2))

attach(V)

plot(time,Rpos,pch=15,col="red",ylim=c(0,max(Rpos)), main="Model fitting",ylab="responses")

points(time,Rneg,pch=16,col="green")

points(time,Neutral,pch=17,col="blue")

res=optim(par=par,llv,time=time,x=cbind(Rpos,Neutral),m=40,control=c(fnscale=-1,maxit=10000),hessian=T)

cat("code convergence :",res$convergence,"\n")

cat("paramètres :",res$par,"\n")

#Mise en page des résultats

if(det(res$hessian)!=0)

{

S=-solve(res$hessian)

se=sqrt(diag(S))

sigma=sqrt(diag(diag(S),7,7))

if(det(sigma)!=0)

{

sm1=solve(sigma)

Mcor=sm1%*%S%*%sm1

Mcor=data.frame(Mcor)

names(Mcor)=rownames(Mcor)=c("ka","aa","ba","kc","ac","bc","t0")

cat("Correlation Matrix estimate\n-----------------------------------\n")

print(Mcor)

}

else cat("sigma singular. No correlation matrix printed\n")

S=data.frame(S)

names(S)=rownames(S)=c("ka","aa","ba","kc","ac","bc","t0")

# cat("Variance-Covariance Matrix estimate\n-----------------------------------\n")

# print(S)

}

else cat("singular hessian. No Variance-covariance matrix printed\n")

tabres=data.frame(cbind(res$par, se,res$par-1.96*se,res$par+1.96*se))

names(tabres)=c("param","se","lower","upper")

rownames(tabres)=c("ka","aa","ba","kc","ac","bc","t0")

tabres

ka=res$par[1];aa=res$par[2];ba=res$par[3]

kb=res$par[4];ab=res$par[5];bb=res$par[6];t0=res$par[7]

u=40*(ka-(ka-aa)/(1+exp(-ba*(time-t0)))+aa)

v=40*(ab+(kb-ab)/(1+exp(-bb*(time-t0))))

lines(time,u,col="red")

lines(time,v,col="blue")

lines(time,40-u-v,col="green")

abline(v=t0,col="blue",lty="dashed")

text(1.05*t0,0.05,"t0",col="blue")

plot(u,Rpos-u,pch=15,col="red",main="Residuals vs fit",xlab="fit",ylab="residuals")

points(v,Neutral-v,pch=16,col="blue")

points(40-u-v,Rneg-40+u+v,pch=17,col="green")

abline(0,0)

detach(V)

par(mfrow=c(1,1))

tabres

}
