# Supplementary material for: Poor adult nutrition impairs learning and memory in a parasitoid wasp
Source: Sci Rep. 2021 Aug 10;11:16220. doi: 10.1038/s41598-021-95664-6 (PMC8355316; doi:10.1038/s41598-021-95664-6)
Supplement: Supplementary file 2 — Supplementary Information 2. [file 41598_2021_95664_MOESM2_ESM.docx]

**Poor adult nutrition impairs learning and memory in a parasitoid wasp**

Hossein Kishani Farahani^1^*, Yasaman Moghadassi^2^, Jean-Sebastien Pierre^3^, Stéphane Kraus^4^, Mathieu Lihoreau^4^*

1. *Equipe Recherches Agronomiques, Agronutrition, Carbonne, France*
2. *Department of Plant Protection, Faculty of Agriculture and Natural Resources, University of Tehran, Karaj, Iran*
3. *University of Rennes 1, UMR-CNRS 6553 EcoBio, Avenue du Général Leclerc, Campus de Beaulieu, 35042 Rennes Cedex, France.*
4. *Research Center on Animal Cognition (CRCA), Center for Integrative Biology (CBI); CNRS, University Paul Sabatier – Toulouse III, France*

* Corresponding authors:

[H.kishani@agro-nutrition.fr](mailto:H.kishani@agro-nutrition.fr)

Equipe Recherches Agronomiques, Agronutrition Co., Carbonne, France

Phone : +33781944086

mathieu.lihoreau@univ-tlse3.fr

Research Centre on Animal Cognition, UMR 5169 CNRS, University of Toulouse III, France, phone: +33 6 33 43 57 06

**Running Title: nutrition quality and adults memory**
